# Gamma/delta T cells as cellular vehicles for anti-tumor immunity

**DOI:** 10.3389/fimmu.2023.1282758

**Published:** 2024-01-11

**Authors:** Chelsia Qiuxia Wang, Pei Yu Lim, Andy Hee-Meng Tan

**Affiliations:** ^1^ Immune Cell Manufacturing, Bioprocessing Technology Institute (BTI), Agency for Science, Technology and Research (A*STAR), Singapore, Singapore; ^2^ Food, Chemical and Biotechnology Cluster, Singapore Institute of Technology (SIT), Singapore, Singapore

**Keywords:** γδ T cell, Gamma/delta T cell, chimeric antigen receptor (CAR), anti-tumor immunity, cancer immunotherapy, Unconventional T cells, non-HLA-restricted T cells, cellular immunotherapy

## Abstract

Adoptive cellular immunotherapy as a new paradigm to treat cancers is exemplified by the FDA approval of six chimeric antigen receptor-T cell therapies targeting hematological malignancies in recent years. Conventional αβ T cells applied in these therapies have proven efficacy but are confined almost exclusively to autologous use. When infused into patients with mismatched human leukocyte antigen, αβ T cells recognize tissues of such patients as foreign and elicit devastating graft-versus-host disease. Therefore, one way to overcome this challenge is to use naturally allogeneic immune cell types, such as γδ T cells. γδ T cells occupy the interface between innate and adaptive immunity and possess the capacity to detect a wide variety of ligands on transformed host cells. In this article, we review the fundamental biology of γδ T cells, including their subtypes, expression of ligands, contrasting roles in and association with cancer prognosis or survival, as well as discuss the gaps in knowledge pertaining to this cell type which we currently endeavor to elucidate. In addition, we propose how to harness the unique properties of γδ T cells for cellular immunotherapy based on lessons gleaned from past clinical trials and provide an update on ongoing trials involving these cells. Lastly, we elaborate strategies that have been tested or can be explored to improve the anti-tumor activity and durability of γδ T cells *in vivo*.

## 1 Introduction to γδ T cells

Recent advances in genomic editing of cells (1–3) have propelled cellular immunotherapy as a new paradigm to treat cancers, which is rapidly gaining traction with the FDA approval since 2017 of six therapies involving T cells engineered with different chimeric antigen receptors (CARs) targeting primarily B cell malignancies [summarized in (4, 5)]. These approved therapies, and many others undergoing investigation in pre-clinical studies and clinical trials, have largely utilized conventional αβ T cells which are limited to autologous applications. If infused in a recipient patient with mismatched human leukocyte antigen (HLA), αβ T cells will recognize as foreign and attack the patient’s tissues that results in potentially life-threatening graft-versus-host disease (GvHD). One approach to circumvent the occurrence of GvHD is to use innate or innate-like immune cells such as γδ T cells, which possess characteristics rendering them appropriate for allogeneic therapy.

**Figure 1:**
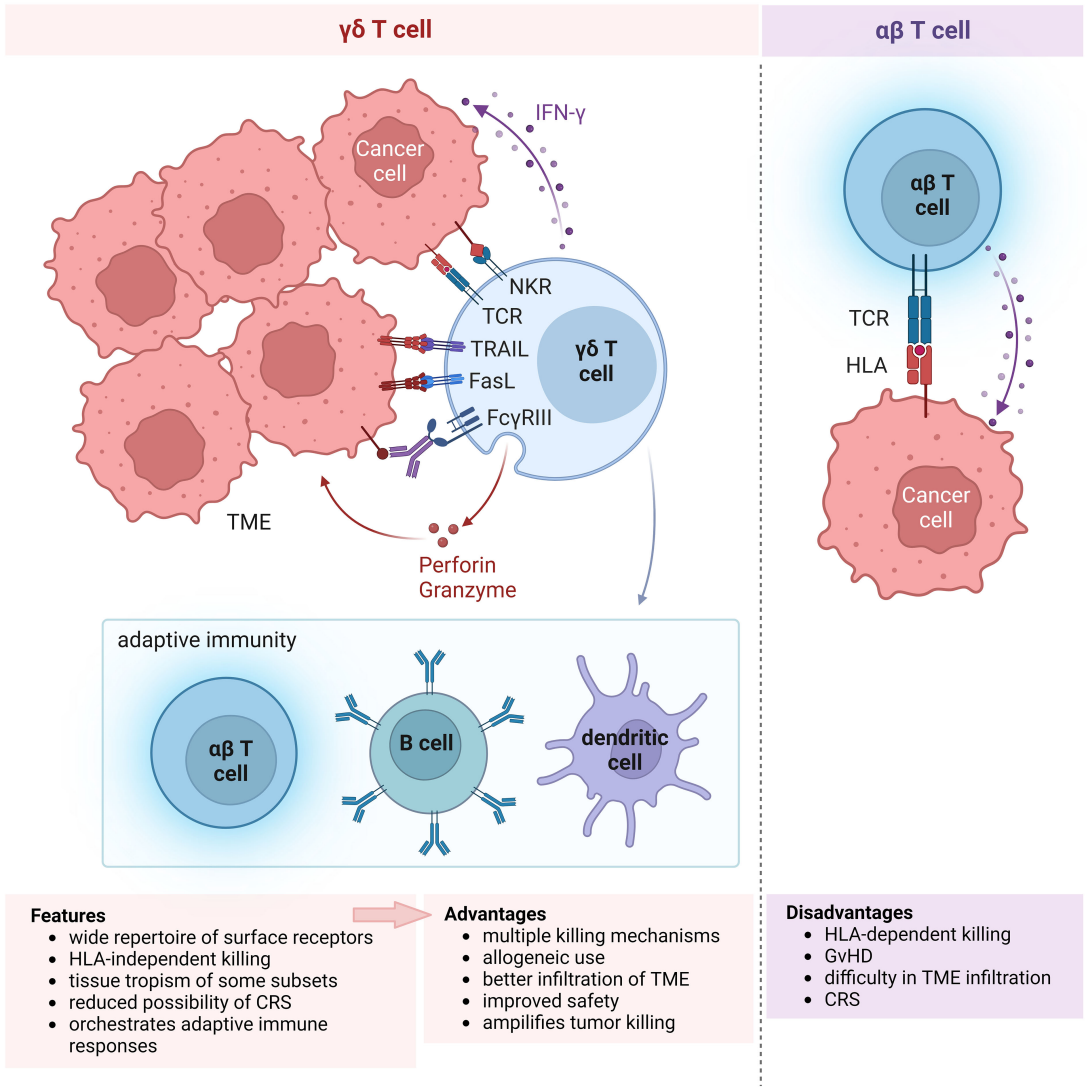
Features of γδ T cells propelling their choice for use in cellular immunotherapy. Schematic showing the multitude of receptors expressed on γδ T cells and their anti-tumor killing mechanisms (left), in comparison with αβ T cells (right). The wide repertoire of surface receptors confer the advantage of multiple killing mechanisms that can be elicited by γδ T cells. Moreover, their killing mechanism is independent of HLA, unlike αβ T cells, allowing for allogeneic use in cell therapy. Activation of γδ T cell killing results in engagement of the adaptive immune response, resulting in the amplification of anti-tumor cytotoxicity. Other features not illustrated in the diagram include tissue tropism of certain, e.g. Vδ1, γδ T cell subsets and the reduced possibility of CRS, presumably leading to better infiltration of TME and improved safety. All of these features of γδ T cells (bottom left) that provide advantages over αβ T cells (bottom right) for use in allogeneic immune cell therapy are summarized in the boxes below. NKR, natural killer receptor; TCR, T cell receptor; TRAIL, tumor necrosis factor (TNF)-related apoptosis-inducing ligand; FasL, Fas ligand; IFN, interferon; TME, tumor microenvironment; HLA, human leukocyte antigen; GvHD, graft-versus-host disease; CRS, cytokine release syndrome.

γδ T cells represent a small population of total leukocytes in umbilical cord blood (UCB) and peripheral blood (PB), comprising approximately 0.0045-0.035% of UCB and 0.5-5% of PB (6–9). Despite their low abundance, these cells play crucial roles in immune defense against bacterial and viral infections, as well as in immune surveillance of cancer. γδ T cells are poised to recognize intracellularly stressed cells, such as infected and tumor cells, and respond by directly eliminating such cells (10). The infected and tumor cells convey their intracellular stress to γδ T cells via a myriad of molecules. γδ T cells sense these dysfunctional cells by recognizing tumor-associated metabolic byproducts such as butyrophilins (BTNs) on tumor cells in the peripheral circulation or stress-associated proteins like MHC class I-related chain A or B (respectively MICA or MICB) upregulated on stressed cells in both PB and tissues. Engagement of these ligands by their receptors on γδ T cells activate direct killing mechanisms via granzyme B and perforin rapidly without prior exposure to pathogen- or tumor-associated antigens (**Figure 1**). They also stimulate secretion of effector molecules such as interferon (IFN)-γ and tumor necrosis factor (TNF)-α.

There are several advantages in employing γδ T cells for immunotherapy (**Figure 1**). Firstly, γδ T cells express a wide repertoire of cell surface receptors conferring the ability to broadly recognize a diversity of tumor ligands and thereby target multiple tumor types, unlike HLA-restricted tumor recognition by αβ T cells. This is particularly useful for tumors which have downregulated HLA class I expression to evade immune recognition by αβ T cells. Secondly, the cytotoxic function of γδ T cells is therefore activated independently of HLA which drastically reduces their chance of provoking GvHD and allows for their allogeneic use. Thirdly, γδ T cells are the major early producers of pro-inflammatory IFN-γ which triggers their anti-tumor response and orchestrates αβ T, B and dendritic cells in a cascade of adaptive immune responses that further amplify tumor killing (11). The cross-talk between γδ T cells and other immune cells are described in a recent review (12). Such interactions in the tumor microenvironment (TME) allow γδ T cells to shape its immediate environment into a tumor-suppressing one. Moreover, γδ T cell subtypes characterized by certain rearrangements of their γδ T cell receptor (TCR) intrinsically populate specific tissues, namely skin, large intestine, spleen and liver. It is thought that such tissue tropism may enhance the capacity of γδ T cell subtypes to infiltrate the TME of diverse solid tumors consisting tissues which are the physiological habitats for the respective cell subtypes. Furthermore, as engineered γδ T cells exhibit similar anti-tumor efficacy but generally secrete lower levels of cytokines compared with their similarly modified αβ counterparts, γδ T cell therapy harbors a potentially lower risk of cytokine release syndrome (CRS) (13–15).

In this review, we summarize fundamental concepts underlying the biology of γδ T cells, as well as recent developments related to their role in cancer prognosis and survival revealed by multiple lines of research evidence which will be elaborated in the following sections. We discuss the gaps in knowledge that can improve ways to harness γδ T cells for cellular immunotherapy. We also take stock of the current outlook of clinical trials relating to γδ T cell therapies that have been carried out thus far and discuss what we can learn from these trials. Lastly, we review current or propose new strategies to improve the anti-tumor efficacy of γδ T cell therapies.

## 2 γδ T cells: what are the gaps to be filled?

### 2.1 Refinement of γδ T cell subtypes and their associated ligands

**Figure 2:**
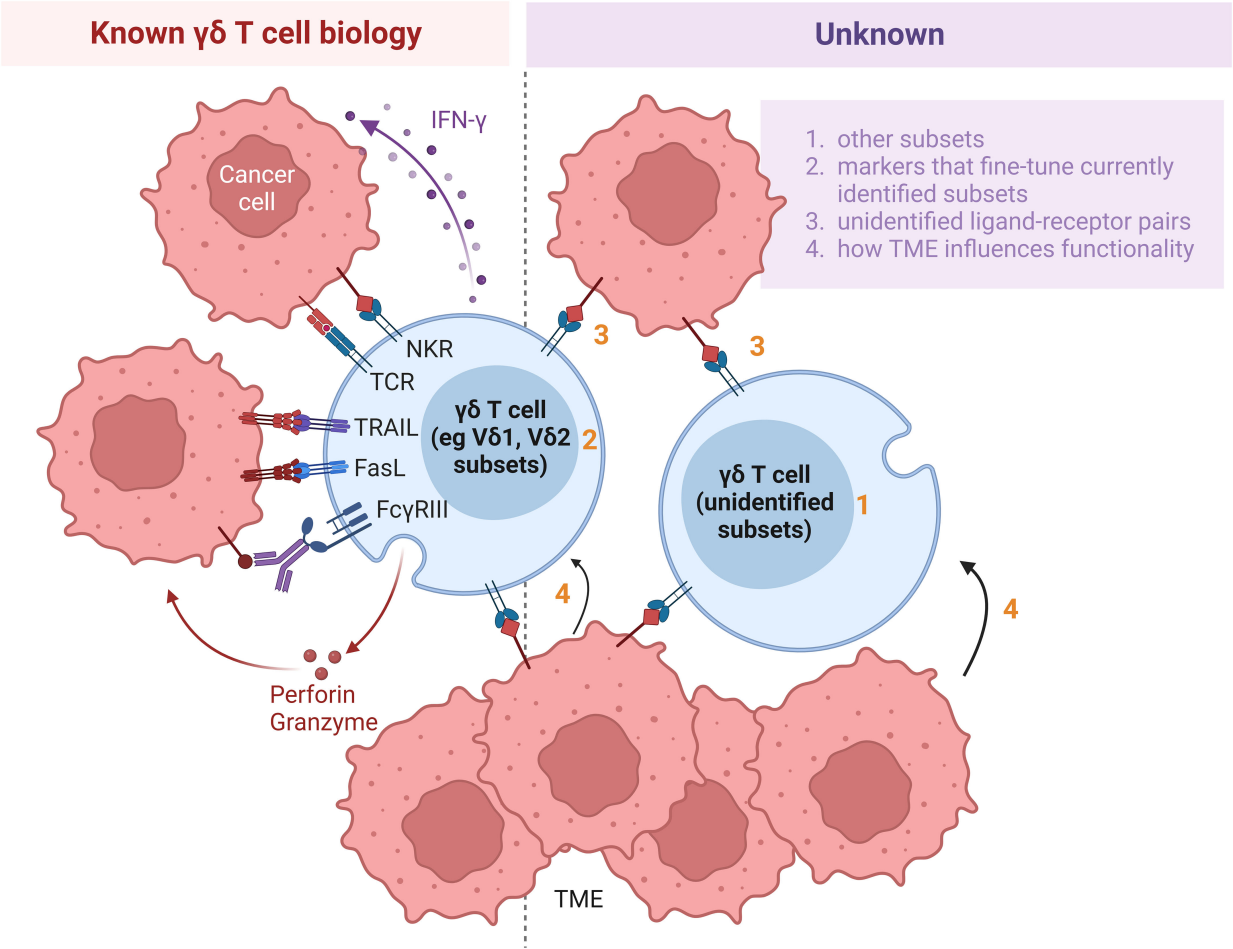
Hurdles impeding application of γδ T cells for cell therapy. Whilst increasing amount of information is being discovered about γδ T cell biology (left panel), much remains to be further elucidated (right panel). Firstly, various subsets, including Vδ1 and Vδ2, have been identified. However, presence of substantial Vδ1 and Vδ2 populations suggest that continuous identification of additional subsets is warranted (point 1). Secondly, within each subset, the γδ T cells can be delineated by additional markers, such as CD56, CD27 and NKG2A. Identification of such additional markers will yield added insight into the functional roles of γδ T cells (point 2). Thirdly, while several molecules have been identified as ligands to the multitude of receptors on γδ T cells, many ligands recognized by TCRγδ or other surface receptors remain currently unidentified (point 3). Furthermore, the influence of the TME on the functions of γδ T cells has been established, but the exact conditions which tune γδ T cells towards pro- versus anti-tumor subsets are not yet well defined (point 4). Research delving to uncover the unknown aspects of γδ T cell biology and their interactions with cells in the TME will improve the effectiveness of adoptive transfer of γδ T cells in immunotherapy. NKR, natural killer receptor; TCR, T cell receptor; TRAIL, tumor necrosis factor (TNF)-related apoptosis-inducing ligand; FasL, Fas ligand; IFN, interferon; TME, tumor microenvironment.

Human γδ T cells can be divided into several subtypes, including Vδ1 and Vδ2 subtypes based on their expression of TCRδ chain variant, contrasting with murine γδ T cell subsets which are categorized according to their γ chain expression. While Vδ2 cells are predominantly found in blood circulation, Vδ1 cells are localized mainly in mucosal epithelial tissues. There also exist less well studied subtypes such as Vδ3 cells that reside in the liver. Regardless of their subtype based on TCRδ chain variant expression, γδ T cells can be distinguished in terms of functional potency based on their expression of cell surface receptors, including CD56 (16, 17), NKG2A (18), the SCART scavenger receptors (SCART1 and SCART2) (19), CD27 (20) and CD161 (21), signatures of which correlate with cytokine secretion and anti-tumor cytotoxicity. Interestingly, Vδ1 and Vδ2 subtypes can each be functionally differentiated by the expression of CD56. While CD56^+^ Vδ2 T cells have greater anti-tumor effector function compared with their CD56^-^ counterparts, the opposite is observed of Vδ1 T cells for which positive expression of CD56 is associated with lower anti-tumor potency. It should be noted that the latter finding was based on tumor-infiltrating Vδ1 T lymphocytes derived from a single patient and hence requires further validation. Delineation of the spectrum of cytotoxic properties within each γδ T cell subset will yield added insight into the functional roles of γδ T cells (**Figure 2**, right, points 1 and 2).

Binding of their TCR ligands activates γδ T cells to secrete IFN-γ, TNF-α and other cytotoxic effector molecules that act against tumor cells (22) (**Figure 2**, left). The activated γδ T cells also secrete granzyme B and perforin which aid in their cytolytic function. While many ligands remain currently unidentified (**Figure 2**, right, point 3), metabolites of the isoprenoid pathway, also known as phosphoantigens, or pharmacological agents that promote their accumulation have been found to efficiently activate and expand Vγ9Vδ2 T cells. Physiologically, upregulation of the mevalonate pathway in tumor cells results in the accumulation of phosphoantigens, such as isopentenyl pyrophosphate, which induce conformational changes of BTN3A1 in these cells (23). In turn, such a conformational change mediates interactions between BTN2A1 and BTN3A1 and leads to the subsequent binding of TCR Vγ9 to BTN2A1. An example of a synthetic phosphoantigen that has been assessed in clinical trials is bromohydrin pyrophosphate (BrHPP). Identification of *de novo* biomolecules that can preferentially stimulate other γδ T cell subsets will facilitate their *ex vivo* and *in vivo* expansion for therapeutic purpose. In addition, Vδ1 T cells can recognize ephrin receptor A2 (EphA2) (24) and MHC-related protein 1 (25), while Vδ3 T cells are activated by annexin A2 (26) on tumor cells and Vδ5 T cells bind endothelial protein C receptor (EPCR) on cytomegalovirus-infected and epithelial tumor cells (27) through their respective TCRs. Due to the tissue tropism of non-Vδ2 T cells, the ligand-receptor recognition pathways involved in the activation of non-Vδ2 T cells presumably play a more important role in γδ T cell activation in the context of solid tumors. Apart from TCR ligands, ligands induced on epithelial and tumor cells via stress or structural damage, including MICA, MICB and UL16-binding proteins (ULBPs), are recognized by NKG2D on both intraepithelial Vδ1 and circulating Vδ2 cells (14). The aforementioned ligands, among others, could be engineered in feeder cells to support *ex vivo* expansion of γδ T cells to attain clinically relevant numbers of γδ T cells which are estimated to be 10^8^ to 10^11^ cells per infusion.

Although most γδ T cell subsets exhibit cytotoxicity against tumor cells, there exist pro-tumorigenic interleukin (IL)-17-producing (28) and PD-L1-overexpressing γδ T cells (29). These broadly termed regulatory γδ T cells (γδ Tregs) antagonize the therapeutic efficacy of cytotoxic γδ T cells and therefore suppress host immune responses (30). Interestingly, prior exposure or not to ligands during development in the murine thymus programs the effector fate of γδ T cells into respectively IFN-γ or IL-17-producing cells (31). The divergent roles of γδ T cell subsets in anti-tumor immunity have to be carefully delineated in order for their innate properties to be harnessed for immunotherapy.

### 2.2 Association of γδ T cells with prognosis and survival outcomes

#### 2.2.1 γδ T cells are frequently associated with positive prognosis and survival

Notwithstanding their dual nature imprinted by thymic development, tumor-infiltrating or circulating γδ T cells are generally correlated with positive clinical outcomes or prognoses (32). Evidence from representative studies on various tumor types are described in this section (**Table 1**). For example, intratumoral Vδ1 T cells harvested from melanoma patients exhibited convincing anti-tumor function *in vitro* and when infused into patients (17). Intratumoral Vδ2 cell frequencies were found to correlate inversely with the stage of melanoma disease, with high Vδ2 frequencies observed in patients lacking cancer metastases and negligible frequencies in patients bearing advanced stage and metastatic melanomas (33). Increased intratumoral infiltration of γδ T cells was associated with overall survival benefit of gastric cancer patients (34). Moreover, Wu et al. reported that Vδ1^+^ cells were more abundant within triple-negative breast cancer (TNBC) vis-à-vis paired healthy tissues, especially when the cancer is in remission (44). Another study by Janssen et al. showed that the predominant population in TNBC was Vδ2^-^ cells eliciting a proinflammatory rather than an IL-17-expressing signature (35). Interestingly, their tumor reactivity is prescribed by the diverse TCRγ and TCRδ chains and less characterized by the more “generic” anti-tumor response achieved via innate receptors such as NKG2D. Whichever the case, these findings support the observation that higher γδ T cell infiltration correlated with better survival of TNBC patients (45). Contrary to a prior study reporting the polarization of IL-17-producing Vδ1^+^ T cells that promote colorectal cancer (CRC) pathogenesis (28), a recent study by Meraviglia S et al. found that tumor-infiltrating γδ T cells expressing the TCRGV9-encoding gene were not the major producers of IL-17 in the CRC TME and their higher frequencies were associated with significantly longer disease-free survival rate (36). Notably, the latter study provided indirect evidence that mediators secreted by CRC cancer stem cells likely inhibited γδ T cell function in TME.

**Table 1:** Association of tumor-infiltrating and circulating γδ T cells with prognosis or survival of patients with different cancers.

Cancer type	TILs or circulating lymphocytes	γδ T cell subtype	References
Studies supporting positive correlation with prognosis or survival			
melanoma*	TIL	Vδ1^+^, Vδ2^+^	(17, 33)
gastric cancer	TIL	unknown	(34)
breast cancer*	TIL	Vδ1^+^, Vδ2^-^	(35)
colorectal cancer*	TIL	Vγ9Vδ2	(36)
prostate cancer	circulating	Vγ9Vδ2	(37)
acute myeloid leukemia, acute lymphoblastic leukemia	circulating	Vδ1^+^	(38)
lymphoma	circulating	Vγ9Vδ2	(39)
B cell chronic lymphocytic leukemia	circulating	Vδ1^+^	(40)
Studies supporting negative correlation with prognosis or survival			
breast cancer*	TIL	Vδ1^+^ Tregs	(41)
pancreatic ductal adenocarcinoma	TIL	Vγ9Vδ2^-^	(29)
colorectal cancer*	TIL	Vδ1^+^ Tregs	(28)
squamous cell carcinoma	TIL	Vδ1^+^ and Vδ2^+^ Tregs	(42)
melanoma*	circulating	Vδ1^+^	(43)

Asterisks (*) highlight cancer types in which γδ T cells are associated with both positive and negative prognosis or survival. TIL, tumor-infiltrating lymphocytes; Tregs, regulatory T cells.

Late-stage prostate cancer patients who were treated with zoledronate and IL-2 had superior clinical outcomes compared with zoledronate alone, as the former combination resulted in greater frequencies and more pronounced activation of peripheral γδ T cells (37). Combined zoledronate and IL-2 therapy elevated populations of γδ T cells bearing effector memory (T_EM_) and terminally differentiated phenotypes (T_EMRA_), with concomitant decrease in cell populations of naïve (T_naïve_) and central memory (T_CM_) phenotypes in all seven patients examined (37).

Separately, a long-term study demonstrated enhanced leukemia-free and overall survival of patients who received allogeneic hematopoietic stem cell transplantation for treatment of acute myeloid leukemia (AML) or acute lymphoblastic leukemia (ALL) when their levels of donor-derived, circulating and predominantly Vδ1^+^ γδ T cells were high (38). γδ T cells which were responsive to proliferative stimulation by pamidronate and low-dose IL-2 contributed to effective anti-lymphoma responses *in vivo* while lack of γδ T cell proliferation correlated with poor objective tumor responses (39). Disease progression in patients suffering from B cell chronic lymphocytic leukemia was associated with low numbers of circulating Vδ1^+^ cells. Reciprocally, patients who had higher Vδ1^+^ cell counts maintained stable disease (40). Taken together, these studies strongly suggest that γδ T cells exert cytotoxic effects against majority of cancer types.

#### 2.2.2 γδ T cells are occasionally associated with negative patient prognosis and survival

As earlier alluded, certain γδ T cell types are known to be tumor-promoting (**Table 1**) given pre-programming of different functional subsets during development and dependence on tumor context in activating selective subsets (30). For instance, Vδ1^+^ Tregs were found to be the dominant tumor-infiltrating lymphocyte (TIL) population in breast cancer tissues examined in 11 patients (41). These Vδ1^+^ Tregs, most being subsequently identified to express CD73, potently suppressed dendritic cell maturation and function, as well as cytokine secretion by CD4^+^ helper T cells and CD8^+^ effector T cells (46). The inhibitory function of Vδ1^+^ Tregs can be abrogated by Toll-like receptor 8 ligand engagement to enhance anti-tumor immunity (41). It was observed that γδ T cells infiltrating pancreatic ductal adenocarcinoma (PDA) overexpressed checkpoint ligands PD-L1 and Galectin-9 to directly suppress αβ T cells, hence creating an immunosuppressive TME (29). Vγ9^+^ cells were noticeably absent, implying that TILs were Vγ9^-^Vδ2^-^ cells. The frequency of IL-17-secreting Vδ1^+^ γδ Tregs present in CRC positively correlated with advanced clinicopathological features of the disease (28). These pro-tumorigenic γδ Tregs were shown to promote the migration, proliferation and accumulation of myeloid-derived suppressor cells (MDSCs) via production of IL-17A, IL-8, GM-CSF and TNF-α. In both PDA and CRC, γδ TILs manifested a T_EM_ phenotype, whereas normal healthy tissue counterparts possessed a T_CM_ phenotype (28, 29). Furthermore, patients in the advanced stages of a type of skin cancer called squamous cell carcinoma harbored more Vδ1^+^ and Vδ2^+^ IL-17-producing γδ T cells in contrast to those in the early stages of cancer which had more IFNγ-producing cells (42). Elevated frequencies of PB Vδ1^+^ T cells in patients with metastatic melanoma were correlated with poorer clinical prognoses, unlike those of PB Vδ2^+^ counterparts which lack association (43).

While human γδ Tregs have been less studied than their murine counterparts (47), it is recognized that pro-tumorigenic γδ Tregs do not exert direct effects on tumor cells but are able to shape the TME via other cell types to become an immune suppressive one, thereby promoting oncogenic progression. Collectively, the aforementioned studies, albeit non-exhaustive, serve as a timely reminder of the opposing roles that γδ T cell subsets play in tumor immunity.

### 2.3 The gaps to fill for the roles of γδ T cells in anti-tumor immunity

In some studies, γδ T cells were not clearly distinguished based on subsets defined by γδ TCR usage, which could affect the interpretation of results, since the anti-tumor properties of different subsets and even populations within the same subset vary with tumor context. To account for population variations going forward, researchers should proactively include γδ T cell subset analyses in their studies. Investigating the effector phenotypes of γδ T subsets offers important insight into their recruitment patterns to tumor sites (33, 42). While identifying the wide range of ligands recognized by γδ T cells continues to pose a challenge to researchers, of greater pertinence is the choice of a specific antigen or antigens that can be used either for *ex vivo* activation and expansion of γδ T cells or direct administration to expand the cells *in vivo*. This is exemplified by the use of BrHPP or zoledronate to expand Vγ9Vδ2 T cells. Furthermore, whether γδ T cells play tumor-suppressive or promoting roles in a particular cancer type is possibly influenced by the specific TME. This can be assessed *in vitro* by co-incubating γδ T cells with supernatants derived from the culture of specific cancer cell types (36, 42). Whether γδ T cells are associated with good or poor prognosis for the same cancer type, such as breast cancer, CRC or melanoma (**Table 1**) may be dependent on the stage of cancer (42). Clearly, identification of specific molecules secreted by cancer cells in the culture supernatant that impact the “fate commitment” of γδ T cells will shed light on possible mechanisms educating the pro- or anti-tumor behavior of these cells in a given TME. Further insights into the interaction between γδ T cell biology and the TME will inform strategies of employing γδ T cells as an effective oncotherapy (**Figure 2**, right).

## 3 Harnessing γδ T cells for anti-tumor immunotherapy

### 3.1 Lessons learnt from past clinical trials

Several γδ T cell immunotherapy clinical trials have been carried out. In **Table 2**, we focus on summarizing the completed and on-going γδ T cell immunotherapy clinical trials that specifically utilized direct γδ T cell administration to provide an overview of their status, phase of trial, types of cells administered, target cancer types, and their clinical outcomes. From the accumulating number of clinical trials, we have gained invaluable insight and herein discuss the important lessons we can learn from these trials. We also put forth several strategies to advance γδ T cell immunotherapy.

**Table 2:** Ongoing and past clinical trials involving direct cellular administration of unmodified and modified γδ T cells, including study outcome (if available).

ClinicalTrials.gov Identifier/reference	Status	Cell type(s) infused	Donor source	Cell source	Modification of cells, if applicable	Trial phase	Condition/disease	Outcome
(48)	Completed	Enriched in Vγ9Vδ2 T cells (Innacell™; single BrHPP stimulation followed by 2-week expansion in presence of IL-2 *in vitro*); infused with IL-2	Autologous	PB	nil	1	Metastatic RCC	n = 10 Efficacy 6 SD: 60% 4 PD: 40% PFS: 25.7 weeks (5-111 weeks) Safety and toxicity DLT: 1 out of 3 patients treated at 8 x 10^9 cells
(49)	Completed	Activated by 2-methyl-3-butenyl-1-pyrophosphate and expansion in the presence of IL-2 until day 14	Autologous	PB	nil	Not applicable	Advanced RCC	n=7 Efficacy 3 PR: 43% Safety and toxicity No serious adverse events observed.
(50)	Completed	Expanded using IL-2 and zoledronate	Autologous	PB	nil	1	NSCLC	n=10 Efficacy 3 SD: 30% 5 PD: 50% Safety and toxicity No serious adverse events observed.
(51)	Completed	Enriched in Vγ9Vδ2 T cells (zoledronate stimulation followed by 2-week expansion in presence of IL-2 *in vitro*); infused with zoledronate	Autologous	PB	nil	1	Breast cancer, cervical cancer and other solid tumors	n=18 Efficacy 1 CR: 6% 2 PR: 11% 3 SD: 17% PR and CR achieved with co-treatment. Safety and toxicity No DLT observed.
**NCT02418481**	Completed	γδ T cells with or without DC-CIK cells	Autologous	PB	nil	1 & 2	Breast cancer	
**NCT02425735** (52)	Completed	Vγ9Vδ2 T cells with or without DC-CIK cells	Autologous	PB	nil	1 & 2	Hepatocellular liver cancer (including CCA)	1 case study published (allogeneic). Efficacy Positively regulated peripheral immune functions of the patient, depleted tumor activity, improved quality of life, and prolonged his life span. Safety and toxicity No adverse effects.
**NCT02425748**	Completed	γδ T cells with or without DC-CIK cells	Autologous	PB	nil	1 & 2	Non small lung cancer (without EGFR mutation)	No published results.
**NCT03180437** (53)	Completed	Vγ9Vδ2 T cells with or without IRE surgery	Allogeneic	PB	nil	1 & 2	Locally advanced pancreatic cancer	n=62 Efficacy Median OS: 14.5 months compared to 11 months without γδ T infusion Median PFS: 11 months compared to 8.5 months without γδ T infusion Safety and toxicity 14 serious adverse events (grade 3 and 4) observed that were likely due to IRE treatment and not γδ T cells
**NCT03183206, NCT03183219, NCT03183232** (54)	Completed	Vγ9Vδ2 T cells expanded using zoledronate, IL-2, IL-15 and vitamin C for 12-14 days	Autologous	PB	nil	1 & 2	Breast cancer, liver cancer and lung cancer, respectively	n=132 Efficacy 18 patients (13.6%) showed response and prolonged survival Median OS (liver cancer patients): 23.1 months compared to 8.1 months in control group Median OS (lung cancer patients): 19.1 months compared to 9.1 months in control group Safety and toxicity No significant adverse events (immune rejection, GvHD or CRS) observed.
**NCT03790072** (55)	Completed	Ex vivo expanded Vγ9Vδ2 T cells (OmnImmune^®^) using zoledronate and IL-2	Allogeneic (matched or haploidentical family donors)	PB	nil	1 & 2	AML	n=7 Efficacy 1 CR: 14% 1 SD: 14% (eventually progressed) 1 MLFS: 14% Safety and toxicity No DLT and significant adverse effect (GvHD or neurotoxicity) observed. 1 patient suffered possible grade 1 CRS.
**NCT04696705**	Recruiting	Ex-vivo expanded γδ T cells	Allogeneic (blood-related donor)	PB	nil	Early phase 1	NHL, PTCL	No published results.
**NCT04702841**	Recruiting	CAR γδ T cells	Autologous	PB	CD7 CAR	Early phase 1	R/r CD7^+^ T cell-derived malignant tumors	No published results.
**NCT03533816**	Recruiting	Expanded/activated γδ T cell, followed by depletion of αβ T-cells (INB-100)	Allogneneic (haploidentical donors)	PB	nil	1	AML, CML, ALL, MDS	n=7 Efficacy 7 CR: 100% PFS: 2.6 - 36 months Safety and toxicity No DLT observed. All patients experienced low grade (1–2) GvHD
**NCT04165941**	Recruiting	γδ T cells (activated and gene modified) (INB-200)	Autologous	PB	MGMT-gene modified to be drug resistant	1	Glioblastoma multiforme	n=8 Efficacy Cohort 1 (single dose) PFS: 7.4-11.9 months OS: 9.6-17.7 months Cohort 2 (3 doses) PFS: 19.4-23.5 months Safety and toxicity No DLT and serious adverse events (CRS and ICANS) observed. Some grade 1-2 treatment emergent adverse events observed.
**NCT04990063**	Recruiting	Tumor killer cells: mixed cocultures of NK cells & γδ T cells	Autologous	PB	nil	1	Advanced NSCLC	No published results.
**NCT05015426**	Recruiting	γδ T cells (Artificial Antigen Presenting Cell-expanded donor T cells)	Allogeneic	Not stated	nil	1	AML	No published results.
**NCT04735471,** **NCT04911478**	Recruiting	Ex vivo activated and expanded Vδ1 T cells, followed by depletion of αβ T cells (ADI-001)	Allogeneic	PB	Anti-CD20 CAR (3H7-CD8 HTM-BBz)	1	Follicular lymphoma, MCL, MZL, burkitt lymphoma, mediastinal lymphoma, DLBCL, NHL	N=16 Efficacy 6 CR: 38% 1 PR: 6% 2 SD: 13% 5 PD: 31% Safety and toxicity No DLT, GvHD, Grade 3 or higher CRS or ICANS reported.
**NCT05400603**	Recruiting	γδ T cells in combination with dinutuximab, temozolomide, irinotecan and zoledronate (Vδ2 T cells)	Allogeneic	PB	nil	1	R/r neuroblastoma (pediatric)	No published results.
**NCT05653271**	Recruiting	Vδ2 T cells (ACE1831) or ACE1831 and obinutuzumab	Allogeneic	PB	anti-CD20 antibody conjugated	1	B cell lymphoma, NHL, DLBCL, primary mediastinal large B cell lymphoma, MZL, follicular lymphoma	No published results.
**NCT04764513**	Recruiting	Ex vivo expanded γδ T cells (expansion from same donors as HSCT)	Allogeneic	PB	nil	1 & 2	Hematological malignancies after allogeneic HSCT: AML, ALL, MDS, lymphoma	No published results.
**NCT04765462**	Recruiting	Ex vivo expanded γδ T cells (expansion from same donors as HSCT)	Allogeneic	Not stated	nil	1 & 2	Malignant solid tumour	No published results.
**NCT05554939**	Recruiting	CAR γδ T cells	Allogeneic	PB	anti-CD19 CAR	1 & 2	R/r B cell NHL	No published results.
**NCT05886491**	Recruiting	Enriched for Vδ1+ γδ T cells (GDX012) after lymphodepleting chemotherapy (fludarabine/cyclophosphamide)	Allogeneic	PB	nil	1 & 2	AML	No published results.
**NCT03849651**	Recruiting	TCRαβ-depleted hematopoietic cell transplantation with additional memory cell DLI and selected use of blinatumomab	Allogeneic/haploidentical	PB	nil	2	ALL, AML, MDS, NK cell Leukemia, Hodgkin lymphoma, NHL, JMML, CML	No published results.
**NCT05358808**	Recruiting	Vδ2 T cells (TCB-008)	Allogeneic	PB	nil	2	AML	No published results.
**NCT05686538**	Recruiting	Innate donor lymphocyte infusion enriched in NK and γδ T cells	Allogeneic	PB/BM	nil	2 & 3	AML, MDS	No published results.
**NCT05388305**	Recruiting	CAR γδ T cells	Allogeneic	Not stated	anti-CD123 CAR	Not applicable	R/r AML	No published results.
**NCT05302037**	Not yet recruiting	CAR γδ T cells	Allogeneic	PB	NKG2DL-targeting CAR	1	Advanced solid tumours or haematological malignancies	No published results.
**NCT03939585**	Not yet recruiting	NK/γδ T cell-enriched product (donor lymphocytes depleted of TCR-αβ T cells and B cells)	Allogeneic (HLA matched sibling donors or partially matched, related haploidentical donors)	PB	nil	1	Allogeneic stem cell transplant candidate AML, ALL, MDS, MPN, LPD	No published results.
**NCT04806347**	Not yet recruiting	TCRαβ+/CD19+ depleted HSC graft	Allogeneic (closely matched unrelated donors or haploidentical related donors)	PB	nil	1	Blood disease	No published results.
**NCT05664243**	Not yet recruiting	γδ T cells (DeltEx) (INB-400)	Allogeneic	PB	genetically-modified (drug resistance immunotherapy)	1 & 2	Recurrent or newly diagnosed glioblastoma	No published results.
**NCT00562666**	Terminated	γδ T cells	Autologous	PB	nil	1	HCC	No published results.
**NCT05001451**	Terminated (business decision, not related to safety)	Enriched for Vδ1+ γδ T cells (GDX012)	Allogeneic	PB	nil	1	AML	No published results.
**NCT05628545**	Withdrawn (COVID Pandemic)	γδ T cells (GDKM-100)	Allogeneic	Not stated	nil	1 & 2	Advanced HCC	No published results.
**NCT02459067**	Terminated	γδ T cells (ImmuniCell^®^)	Autologous	PB	nil	2	Malignant melanoma, NSCLC, RCC	No published results.
**NCT04700319**	Unknown	CAR γδ T cells	Autologous	PB	CD19/CD20 CAR	Early phase 1	Advanced CD19/CD20^+^ B cell line recurrent or refractory haematological malignancies	No published results.
**NCT04028440**	Unknown	γδ T cells	Autologous	PB	nil	Early phase 1	NHL, r/r B cell NHL, CLL, PTCL	No published results.
**NCT04518774**	Unknown	Ex-vivo expanded γδ T cells	Allogeneic (blood-related donor)	PB	nil	Early phase 1	HCC	No published results.
**NCT02656147**	Unknown	CAR γδ T cells	Allogeneic	Not stated	Anti-CD19-CAR	1	Leukemia, lymphoma	No published results.
**NCT04008381**	Unknown	Ex-vivo expanded γδ T cells	Allogeneic (blood-related donor)	PB	nil	1	AML	No published results.
**NCT04107142**	Unknown	CAR γδ T cells	Allogeneic/haploidentical	PB	NKG2DL-targeting CAR	1	Colorectal cancer, TNBC, sarcoma, NPC, prostate cancer, gastric cancer	No published results.
**NCT02585908**	Unknown	γδ T cells with or without CIK cells	Autologous	PB	nil	1 & 2	Gastric cancer	No published results.
**NCT04796441**	Unknown	CAR γδ T cells	Allogeneic	PB	anti-CD19 CAR	Not applicable	Relapsed AML	No published results.
**NCT03885076**	Unknown	CAR Vδ2 T cells	Autologous	PB/BM	anti CD33 CAR	Not applicable (observational study)	AML (except M3)	No published results.

DC, dendritic cells; CIK, cytokine-induced killer cells; IRE, irreversible electroporation; HSCT, hematopoietic stem cell transplantation; HSC, hematopoietic stem cell; HLA, human leukocyte antigen; PB, peripheral blood; BM, bone marrow; CAR, chimeric antigen receptor; HCC, hepatocellular carcinoma; CCA, cholangiocarcinoma; EGFR, epidermal growth factor receptor; NSCLC, non-small cell lung cancer; RCC, renal cell cancer; r/r, relapsed or refractory; NHL, non-Hodgkin lymphoma; PTCL, peripheral T cell lymphoma; AML, acute myeloid leukemia; CML, chronic myeloid leukemia; CLL, chronic lymphocytic leukemia; ALL, acute lymphoblastic leukemia; T-ALL, T-cell acute lymphoblastic leukemia; TNBC, triple-negative breast cancer; MDS, myelodysplastic syndromes; MPN, myeloproliferative neoplasm; LPD, lymphoproliferative disorders; MCL, mantle-cell lymphoma; MZL, marginal zone lymphoma; DLBCL, Diffuse large B cell lymphoma; NPC, nasopharyngeal carcinoma; JMML, Juvenile myelomonocytic leukemia; EGFR, epidermal growth factor receptor; TAC, T cell antigen coupler; CR, complete response; PR, partial response; SD, stable disease; MLFS, morphologic leukemia-free state; PFS, progression-free survival; OS, overall survival; DLT, dose-limiting toxicity; CRS, cytokine release syndrome; GvHD, graft-versus-host disease; ICANS, immune effector cell-associated neurotoxicity syndrome.

Strategies to utilize γδ T cells for cancer immunotherapy are summarized in recent reviews (56–59). These include the activation or stimulation of endogenous γδ T cells via exogenous aminobisphosphonates and anti-CD3/anti-tumor antigen bispecific antibodies as well as *ex vivo* expansion of peripheral blood-derived γδ T cells (60). Despite their purported capability to target diverse tumor cell types, γδ T cells have performed poorly in clinical trials, yielding largely disappointing clinical outcomes exemplified by low objective tumor response rates and almost no complete responses, with the exception of IN8bio’s trial (NCT03533816) and Adicet Bio’s trial (NCT04735471) which reported 100% and 69% complete responses respectively (59) (**Table 2**; refer to **Supplementary Table** for additional fields of information). Long-term outcome data are currently limited as many of the clinical trials are still on-going and many of them are in the early phases, which focus on establishing safety profile and dose limiting toxicity. Nevertheless, γδ T cell therapy has shown to increase the overall survival and progression-free survival of patients in a limited number of studies (NCT03533816, NCT04165941, NCT03180437, NCT03183206, NCT03183219, NCT03183232) (48, 53, 54), with the longest survival outcomes observed in IN8Bio’s trial in which one patient had progression-free survival for at least 3 years. This is remarkable considering that patients treated in this trial had high-risk AML or failed multiple treatments before receiving γδ T cell therapy. While we can only speculate the reasons why these trials showed exceptional γδ T cell efficacy compared with the rest of the trials, we noted that the therapy targeted hematological malignancies for which patient outcomes are typically more favorable compared with those for solid tumors. In IN8bio’s trial, patients underwent haploidentical bone marrow (BM) transplantation followed by cyclophosphamide treatment prior to γδ T cell infusion. The regime preceding γδ T cell infusion could have synergized with the latter’s therapeutic effects. In Adicet Bio’s trial, γδ T cells were programmed with anti-CD20 CAR which likely increased their efficacy to recognize and kill B lymphoma cells. The company’s proprietary expansion process may also have enriched for the subset of cytotoxic Vδ1 T cells. Other factors to consider are discussed in the following subsections.

#### 3.1.1 Factors affecting tumor-infiltration of γδ T cells must be considered

Even though γδ T cells are one of the major populations found in solid tumors (32), not many studies have extensively characterized their infiltration when human clinical trials are carried out. Of all the completed trials, only Nicol and colleagues reported the migration of γδ T cells after infusion. They observed that the adoptively transferred γδ T cells migrated rapidly to lungs within a few hours before travelling to the liver and spleen. However, only a small number of γδ T cells were found to traffick to tumor sites (51). More studies are needed to understand the infiltration capabilities of adoptively transferred γδ T cells in solid tumors. Knowledge on the phenotypes of γδ T cells that have successfully migrated to tumor sites will also shed light as to why patient outcomes are generally poor for solid tumors compared to hematological malignancies. One can then devise potential solutions to overcome some of the hurdles impeding solid tumor immunotherapy. For detailed discussion on tumor infiltrating γδ T cells and their clinical relevance in cancer patients, we refer readers to other review papers (61, 62). Even if γδ T cells manage to infiltrate solid tumors, another immediate hurdle that they must overcome is the hostile conditions they are subjected to within the TME.

#### 3.1.2 The tumor microenvironment inhibits anti-tumor immune responses

γδ T cells are subjected to signals within the TME, which can drive their differentiation into different functional subsets (63). Cells in the TME comprise of immunosuppressive tumor associated macrophages, MDSCs, cancer-associated fibroblasts and tumor cells themselves, among others. These cells can secrete immunoinhibitory molecules, such as TGF-β (64), which in turn promote the pro-tumorigenic polarization of γδ T cells. In addition, γδ T cells can become exhausted and dysfunctional in the TME of certain tumors. For example, programmed cell death protein 1 (PD-1), LAG-3 and TIM3 were shown to be upregulated in γδ T cells infiltrating multiple myeloma, and together with the increased expression of the cognate ligands on tumor cells, result in their anergy (65, 66).

Cells in the TME can also directly inhibit the anti-tumor cytotoxicity of γδ T cells (67). It has been shown that PDA cells upregulate cyclooxygenase-2 (COX-2) expression in response to IFN-γ and TNF-α secreted by γδ T cells (68). COX-2 leads to an increase in PGE2 in tumor cells as a result of increased enzymatic action. As a consequence of PGE2 binding to their receptors on γδ T cells, TCR signaling is inhibited and this in turn causes the dampening of γδ T cell cytotoxic function. Elevated Cox-2 expression was also observed in breast cancer (69). Recently, it was demonstrated that IL-10 secreted by EBV-transformed lymphoblastoid B cell lines reduced the cytotoxicity of Vγ9Vδ2 T cells (70). In addition, Tregs have been shown to inhibit the proliferation of γδ T cells (71).

The anti-tumor activity of γδ T cells is also highly suppressed by tumor hypoxia in various cancers (72–74). Even if γδ T cells could infiltrate solid tumors, their cytotoxicity can be suppressed by hypoxic conditions in the TME due to apoptosis via PD-1 and reduced expression of NKG2D. In brain tumors, the use of metformin, a repurposed drug that has been shown to elicit an anti-tumor effect (75, 76), reduced hypoxia and rescued the anti-tumor effect of γδ T cells (72). In oral cancers, blockade of PD-1 or targeting hypoxia-inducible factor-1α could also help to overcome tumor hypoxia (73). On the other hand, in the case of breast cancer, cancer cells may also evade detection by γδ T cells by shedding MICA under hypoxia (74). Therefore, strategies for γδ T cells to prevail under TME conditions should be catered for specific tumor types. Taken together, more studies are required to characterize both γδ T and cancer cells, and their interactions in the TME.

#### 3.1.3 Culture conditions during *ex vivo* expansion influences γδ T cell functionality

Besides understanding what happens *in vivo*, the process of *ex vivo* expansion can affect γδ T cell phenotypes and cytotoxicity. Despite the great success achieved by the two trials mentioned earlier in treating liquid tumors, we noted that the clinical outcomes in a study (NCT03790072) that also targeted liquid tumor pale in comparison, with a complete response rate of 14%. The media and/or expansion method employed could have affected the quality, quantity and ultimately the efficacy of γδ T cells produced. Xu et al. examined the effect of γδ T cells grown in the presence of different media supplements and infused into patients on the patients’ overall survival (54). Eighteen patients that were administered with Vγ9Vδ2 T cells grown in their newly formulated media supplemented with zoledronate, IL-2, IL-15 and vitamin C, were found to have better overall survival compared with patients that were administered with γδ T cells grown in media supplemented with zoledronate and IL-2. When expanded with the new formula, the authors obtained higher cell yield and observed less cell death corroborated by RNAseq results showing downregulation in expression of apoptosis-related genes. In addition, there was an increase in Vγ9Vδ2 T cells harboring terminally differentiated effector memory (CD45RA^+^CD27^-^) phenotype which were previously found to express homing receptors such as CCR5 and CXCR3 (77), and a decrease in cell populations with naïve (CD45RA^+^CD27^+^) and central memory (CD45RA^-^CD27^+^) phenotypes, although there were no significant changes in counterparts bearing effector memory (CD45RA^-^CD27^-^) phenotype. The cells also more highly expressed co-stimulatory molecules such as CD80, CD86 and MHC-II. Collectively, these data suggest that appropriate media supplements can prime γδ T cells to migrate to tumor sites and exert cytotoxic effects, thus leading to better clinical outcomes.

#### 3.1.4 γδ T cell subtypes variably affect the clinical outcome

**Figure 3:**
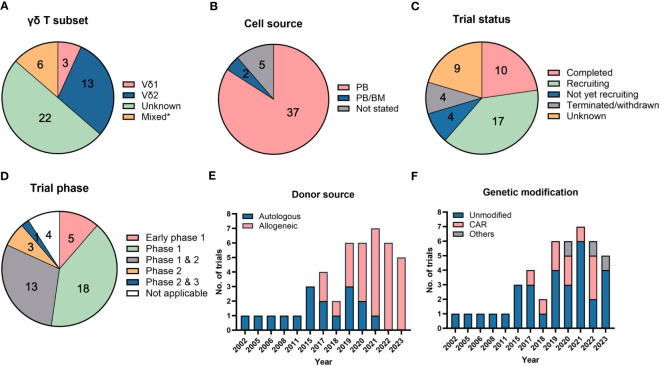
Clinical trials using direct cellular administration of γδ T cells. Pie charts showing the **(A)** γδ T subsets that were infused into patients, **(B)** cell sources from which γδ T cells were obtained, **(C)** trial status and **(D)** different clinical trial phases. *”Mixed” in **(A)** refers to the use of other cell types, namely natural killer (NK), dendritic cell-cytokine-induced killer (CIK) or CIK cells, that were infused together with γδ T cells. “Unknown” in **(A)** refers to trials in which details on γδ T subsets were not available. The category “not applicable” in **(D)** is used for trials without FDA-defined phases, according to clinicaltrials.gov, and includes observational studies. The number of trials in each category is listed within the pie charts in **(A–D)**. Bar graphs depicting the **(E)** donor sources from which γδ T cells are derived and **(F)** types of modifications in γδ T cells. A total of 44 trials were analyzed in **(A–F)**.

Another possible reason for the dismal failure is that a significant proportion of trials focused on harnessing Vγ9Vδ2 γδ T cells for therapy (**Figure 3A**) as they can be readily expanded *ex vivo* to large numbers using zoledronate and IL-2. However, Vγ9Vδ2 cells are naturally abundant in PB and do not typically home to tissues which may partially explain their limited cytotoxic capacity against solid tumors. Vδ1-enriched delta one T (DOT) cells (60), polyclonal γδ T cells comprising multiple subsets or other non-Vδ2 subsets (6) have been or could be explored as potential alternatives that demonstrate greater potency against such tumors. For example, Vγ4^+^ TCRs have been shown to bind butyrophilin like 3 expressed by gut epithelial cells and EPCR expressed mostly by endothelial cells (27, 78) to facilitate immunosurveillance of virus-infected and tumor cells. Such ligands are thought to mediate homing of γδ T cells to and their killing of tumors. Moreover, UCB-derived Vδ2^-^ T cells were shown to be more cytotoxic than their Vδ2^+^ counterparts (8). Other tumor-associated ligands recognized by non-Vδ2 TCRs are summarized in a recent review by Dong R et al. (79) As such, the relative importance amongst the various γδ T cell subsets and which ones should be used in the application for γδ T cell therapy should be considered. In addition, incorporation of a step to specifically deplete pro-tumorigenic subsets prior infusion could improve clinical outcome.

#### 3.1.5 Cell source used for *ex vivo* expansion can influence the properties of γδ T cell product

Clinical trials typically rely on adult PB as a source to harvest and expand γδ T cells with a restricted TCR repertoire (**Figure 3B**). Expansion from UCB will generate “younger” cells equipped with a more polyclonal TCR repertoire able to recognize a broader diversity of tumor ligands but are not yet endowed with distinct homing properties characteristic of adult, tissue-resident γδ T cells (59). In-depth characterization of the functional profiles, such as cytokine secretion and varying TCR affinities towards different ligands, of polyclonal γδ T cells will be important to ascertain advantages of their therapeutic use.

#### 3.1.6 γδ T cell therapy is safe, but its anti-tumor potency requires improvement

The clinical safety of unmodified γδ T cells has been confirmed largely by the paucity of serious adverse events following either dose escalation of aminobisphosphonates and IL-2 to stimulate their *in vivo* expansion in patients or *ex vivo* expansion and subsequent adoptive transfer to patients in multiple trials (80, 81). γδ T cell therapy is relatively safe and accompanied by low-grade adverse events such as fever, fatigue, or gastrointestinal disorder, some of which can self-resolve in a few days (49, 51). However, naturally occurring γδ T cells in most of these trials failed to promote substantial tumor regression and enforce remission, highlighting the need for targeted engineering to (1): instruct commitment of γδ T cells towards cytotoxic and not regulatory lineage and (2) restore their metabolic fitness compromised by the immunosuppressive TME.

Addressing the former challenge would entail utilizing culture conditions and specific antigens to expand and enrich γδ T cells with anti-tumor properties, i.e. cytotoxic T cells, while minimizing or depleting those with pro-tumorigenic properties, i.e. γδ Tregs. In addition, tackling the issues of T cell infiltration and immunosuppressive TME would require strategies such as rejuvenation of T cells via immune checkpoint inhibition. More details are described in section 3.3. Improving anti-tumor potency of γδ T cell therapy is a pressing issue because these relatively newer therapies will no doubt be continuously compared to currently approved CAR-T therapies for lymphoma and myeloma.

### 3.2 Clinical trials involving administration of unmodified or engineered γδ T cells

The strategy to engineer and expand γδ T cells *in vitro* followed by their *in vivo* infusion compared with combinatorial administration of stimulatory γδ T ligands and cytokines to selectively expand these cells *in vivo* likely enables more robust and precise improvement of γδ T anti-tumor efficacy. Here, we summarize in **Table 2** clinical trials which implemented or are in process of implementing a regimen of *ex vivo* expansion followed by infusion of unmodified or CAR-modified γδ T cells into cancer patients, providing evidence that engineered γδ T cells are increasingly preferred to unmodified counterparts for tumor immunotherapy.

Out of the 44 clinical trials in **Table 2**, 10 are completed trials, all of which involved unmodified γδ T cells (**Figure 3C**). Seventeen clinical trials are currently recruiting patients. A broad range of cancers are targeted in these clinical trials with a trend towards trials targeting liquid or blood (24 trials) compared to solid malignancies (21 trials). Among blood cancers, AML is the most common cancer targeted whereas lung, liver and breast cancers are the most commonly targeted solid tumors.

Interestingly, we note that most of these trials utilized PB or BM as their cell sources and there are currently no trials utilizing γδ T cells expanded from UCB and induced pluripotent stem cells (iPSCs) (**Figure 3B**), although there is ongoing work in the field to generate γδ T cells from these sources (8, 82). iPSCs could potentially be an unlimited cell source for allogeneic treatment, which requires a large number of cells for scale-up. iPSCs-derived γδ T cells have been successfully generated and shown to illicit cytotoxicity effect on several cancer cell lines (83).

While majority (91%) of the clinical trials are in their early phases (Phase 1 or 2) (**Figure 3D**), an allogeneic treatment study using TCRαβ/CD19-depleted innate donor lymphocyte infusion has been approved for Phase 2 & 3 clinical trial in 2023 (NCT05686538) (**Table 2**). Besides the clinically proven advantageous safety profile of γδ T cells in not causing GvHD, another reason favoring adoption of allogeneic therapy is the difficulty in recovering and expanding sufficient numbers of autologous γδ T cells of high quality from diseased patients. This is exemplified by trials conducted by Fuda Cancer Hospital, China (NCT03183206, NCT03183219, NCT03183232) which initially planned to use patients’ own PB-derived γδ T cells but the investigators encountered challenges in expanding the cells, prompting them to source cells from allogeneic donors. Similar manufacturing obstacles in some patients were recorded by Vydra et al. when they conducted a trial using autologous γδ T cells (55). In recent years, the number of clinical trials for the allogeneic use of γδ T cells has surpassed those for autologous treatments (**Figure 3E**).

To further improve the specificity and efficacy of γδ T cells for targeting tumors, several strategies have been explored by others, which are discussed in Section 3.3. Equipping γδ T cells with CAR is one of the earliest and most common strategies, given the success witnessed in CAR-modified αβ T cells. Anti-CD7, anti-CD19/20, anti-CD33, and anti-CD123 CAR have been designed to target liquid cancers, while NKG2DL-targeting CAR is constructed for targeting solid tumors. Since 2017, the number of trials conducted with genetically modified γδ T cells has been increasing (**Figure 3F**). As of 2023, there are 10 trials that involved CAR-modified γδ T cells. Besides engineering CAR, γδ T cells have also been modified to be resistant to chemotherapy drug, temozolomide, which is useful to treat glioblastoma using combination therapy (NCT04165941, NCT05664243).

### 3.3 Strategies to augment anti-tumor cytotoxicity of γδ T cells

In this section, we describe how various strategies have been applied or can be adapted to improve anti-tumor efficacy of γδ T cells. Such strategies include those that were used to modify conventional αβ T cells or can complement their therapy via non-genetic engineering approaches. *In vitro* and pre-clinical data, where applicable, are discussed.

#### 3.3.1 Non-genetic engineering approaches

As a first illustration, a bispecific T cell engager (BiTE) antibody construct, AMG 330, administered to leukemic patients yielded encouraging safety and anti-leukemic outcomes (84). A BiTE is a synthetic fusion protein which is designed based on linking the antibody-binding domains of two antibodies. In this example, AMG 330 simultaneously binds CD33 antigen on leukemic blasts and CD3 co-receptor on T cells, placing T cells in close proximity to CD33^+^ leukemic cells and ultimately mediating destruction of the latter by the former cells. In similar fashion, a bispecific tribody which recognizes Vγ9 on γδ T cells and ERBB2 (HER2/neu) on pancreatic cancer cells enhanced γδ T cell cytotoxicity against PDA *in vitro* and *in vivo* (85). Recombinant immunoligands comprising an anti-CD20 single chain variable fragment (scFv) linked to a NKG2D ligand, MICA or ULBP2, activated specific elimination of CD20^+^ but not CD20^-^ lymphoma cells by *ex vivo* expanded Vδ1 and Vδ2 γδ T cells, a therapeutic result which could be further augmented by concurrent agonistic stimulation of the cells with BrHPP (86). Hence, exogenous application of BiTE or similarly designed molecules can be employed in combination with γδ T administration.

Another example obviating non-genetic modification involves programming γδ T cells with various combinations of cytokines to enhance their tumor killing capacity (87). Schilbach and colleagues demonstrated that the combination of IL-2, IL-12 and IL-18 synergize to significantly induce both IFN-γ and TNF-α secretion in the presence of TCR stimulus (88). The increase in TNF-α was observed even in the absence of a TCR signal. The authors also showed that in IL-2/IL-12/IL-18 stimulated γδ T cells, granzyme B and perforin protein expression was upregulated to a similar extent compared to TCR stimulation. Interestingly, the expression of FasL was increased under conditions of IL-2/IL-12/IL-18 stimulation, but not TCR stimulation. Together, these mechanisms mediate the increased anti-tumor killing capacity of cancer cells by the stimulated γδ T cells. More recently, Liu and colleagues showed that γδ T cells pre-treated with a combination of IL-12, IL-18 and IL-21 led to their enhanced inhibition of tumor growth not only *in vitro*, but also *in vivo* after adoptive transfer (89). They showed that such a pre-activation cocktail promoted the proliferation of γδ T cells and their secretion of IFN-γ and TNF-α, which can promote the anti-tumor function of endogeneous CD8^+^ T cells *in vivo*. Therefore, in the cytokine pre-treatment strategy, γδ T cells can be stimulated *ex vivo* by cytokine combinations to boost their anti-tumor activity. Potent cytokine-activated Vδ1^+^ DOT cells have been generated and appears promising for clinical use (60).

Based on newly acquired knowledge on the mechanism of Vγ9Vδ2 T cell activation, agonistic antibodies directed against BTN3A1 and BTN2A1 (90, 91) can be used to heighten sensitivity of tumor cells to γδ T cell killing and offers a promising therapeutic strategy to enhance γδ T cell cytotoxicity. An anti-BTN3A monoclonal antibody (ICT01) is currently in phase 1/2a clinical trial (NCT04243499).

Other possible strategies are targeted at overcoming the immunosuppressive effects of the TME on γδ T cells. For instance, CD137 costimulation using a recombinant CD137L protein was found to reduce the expression of IL-10 receptor, IL-10R1, thereby reducing the sensitivity of the γδ T cells to the immunosuppressive effects of endogenous IL-10 (70).

Interestingly, it was shown that acute systemic β-adrenergic receptor activation was largely responsible for the exercise-augmented mobilization, *ex vivo* expansion and anti-tumor activity of Vγ9Vδ2 T cells from healthy donors (92). Administration of an antagonist inhibiting both β1- and β2-adrenergic receptors abrogated these exercise-induced effects. This finding suggest that β-adrenergic receptors are potential targets to improve the potency of *ex vivo* expanded γδ T cells.

Novel methods of γδ T cell delivery other than the traditional intravenous infusion route could be designed to improve their efficacy in solid tumors. When CAR-T cells were delivered to tumor sites directly using biopolymer scaffolds, they were able to migrate to and kill tumor cells more effectively as compared to systemic delivery method (93). Treatment of glioblastoma has been notoriously challenging due to the difficulty in reaching the blood-brain barrier by immune cells. This could be overcome by stereotactic injection of γδ T cells directly into the brain (94, 95). These direct intratumoral delivery methods could be applied to treat solid tumors that are known to be difficult to infiltrate by immune cells.

Beyond delivering γδ T cells *per se*, it is noteworthy that newer strategies using cell-free extracellular vesicles, such as exosomes, confer a safety advantage over cell-based therapies and have shown promising anti-tumor efficacy. The small size (20-200 nm) of exosomes renders easy infiltration into solid tumor sites and they are resistant to the immunosuppressive TME. Such γδ T cell-derived vesicles were shown to control tumor progression of and elicit anti-tumor responses against Epstein-Barr virus-associated B-cell lymphoma, gastric carcinoma and nasopharyngeal carcinoma (96, 97). These exosomes derived from activated Vδ2 T cells were not only positive for NKG2D, which is responsible for their uptake by tumor cells, but were also positive for FasL and TRAIL, which facilitate their death-inducing properties. More recently, γδ T extracellular vesicles were used as carriers to deliver tumor-associated antigens, and the extracellular vesicles-based cancer vaccines were successful in controlling tumors *in vivo* (98).

#### 3.3.2 Genetic engineering approaches

CAR-modified γδ T cells were first explored as effector cells of tumor-directed immunity in a 2004 study which demonstrated these cells efficiently recognized CAR antigen-expressing neuroblastoma and malignant B cell tumour cells as assessed by their upregulation of CD69 and secretion of IFN-α (99). Consistent with operating multiple mechanisms of cytotoxicity, CD19 CAR γδ T cells were found to exert not only CAR-directed activity against CD19^+^ leukemia cells but also CAR-independent activity against CD19^-^ leukemia cells or cells which have lost expression of CD19 antigen (100), highlighting the advantage of using γδ T vis-à-vis αβ T cells. Although arming γδ T cells with CARs endows tumor specificity, CAR signaling components can be optimized to increase γδ T efficacy against hematological malignancies and solid tumors. Firstly, transduction with second-generation CARs bearing CD3ζ activation domain is known to elicit tonic signaling and exhaustion marked by PD-1 and TIM-3 upregulation in αβ and γδ T cells. Modifying the endodomain of the chimeric co-stimulatory receptors (CCRs) that replace CD3ζ with DAP10 domain in γδ T cells led to effective activation of cytotoxic responses in the presence of CCR-specific stimuli or cognate tumor cells (101). Vδ1 T cells that were genetically modified to express 4-1BB/CD3 CAR targeting the oncofetal antigen glypican-3 and a constitutively secreted form of IL-15 exhibited superior proliferation and anti-tumor activity against hepatocellular carcinoma (HCC) lines and HCC subcutaneously engrafted in immunodeficient mice compared with their non-cytokine secreting counterparts (102). Such armored CAR design which allows release of transgenic cytokine(s) of interest upon CAR signaling had previously been employed successfully in αβ T cells to counteract the inhibitory cytokine milieu of and recruit innate effector cells into the TME (103, 104). Therefore, continued innovation of CAR designs is warranted.

Beyond CAR, introduction of a tumor-specific αβ TCR and the corresponding CD4 or CD8 co-receptor for recognition of HLA-restricted tumor antigen in γδ T cells led to their pronounced cytokine secretion and cytolytic effects against leukemia (105). One obvious drawback using αβ TCR is the requirement for additional CD4 and CD8 co-receptors. The advent of CRISPR/Cas technology has opened new avenues for genetic, including TCR, modification of γδ T cells. Such targeted TCR editing enables controlled replacement of the endogenous TCR with the transgene, thereby allowing for transgene TCR to be expressed at homogeneous, physiological levels on the T cells, and consequently less functional variability compared to virus-mediated transgene integration (106). In this respect, Immatics, a clinical-stage biopharmaceutical company, has entered into a research collaboration and licensing agreement with Editas Medicine, a genome editing company, to advance off-the-shelf adoptive γδ T cell therapy platform. Reciprocally, Vγ9Vδ2 TCR was shown to effectively reprogram both CD4^+^ and CD8^+^ αβ T cells to kill a broad diversity of cancer but not normal cells, and substantially diminished but did not completely abrogate alloreactivity (107). Recently, a clinical stage immune-oncology company, Triumvira Immunologics, developed proprietary T cell Antigen Couplers (TACs) for incorporation in T cells (108). TAC consists of 3 components: a tumor antigen binding domain, a CD3 binding domain which interacts with and co-opts the native TCR and a CD4 co-receptor transmembrane and intracellular domain. When bound to its target antigen, TAC triggers the native TCR signaling cascade by recruiting downstream kinases and thereby activating T cell killing in an HLA-independent manner. TAC-modified αβ T cells are currently undergoing Phase 1 & 2 clinical trials for autologous treatment of HER2^+^ solid tumors (NCT04727151). HER2-targeting TAC-modified γδ T cells are similarly being developed and preclinically evaluated. TAC γδ T cells were observed to exhibit cytotoxicity against tumor xenografts that are resistant to unmodified γδ T cells (109), suggesting modifications of γδ T cells need not be restricted to CAR. However, the safety of their use requires further evaluation.

Despite the established clinical safety profile of unmodified γδ T cells, the enhanced anti-tumor efficacy achieved by modification of γδ T cells may correspondingly increase their off-tumor, on-target toxicity, resulting in undesirable side effects. To address this potential challenge, non-signaling CARs (NSCARs) lacking signaling/activation domains but retaining tumor-specific targeting capability were expressed in γδ T cells. CD5- and CD19-targeting NSCARs significantly elevated the intrinsic, HLA-independent cytotoxicity of γδ T cells against T cell and B cell ALL but expectedly did not enhance the antigen-specific cytotoxicity of αβ T cells (110). An alternative T cell therapy platform involving the concept of a synthetic agonistic receptor (SAR) originally applied in αβ can be potentially extended to γδ T cells. SAR-transduced T cells are directed by an engineered tandem scFv construct (taFv) to antigen-expressing tumor cells in a manner similar to BiTEs (111). The taFv construct comprises two scFvs, one binding the artificial antigen receptor composing an extracellular EGFRvIII domain fused to intracellular T cell-activating domains transduced in T cells and another binding a specific antigen on the surface of cancer cells, thus juxtaposing T and cancer cells. Unlike the BiTE approach which activates pan-T cells, this system specifically activates SAR-transduced T cells and is able to terminate SAR T cells via antibodies clinically approved by FDA should adverse toxicity events arise.

Strategies to improve anti-tumor cytotoxicity of γδ T cells need not be confined to improving recognition of tumor antigens. Other options include boosting the infiltration of γδ T cells into solid tumor by expressing surface proteins that can aid its migration through the extracellular matrix (ECM) surrounding the tumor whilst harnessing the diverse HLA-independent receptors of γδ T cells to target tumors, particularly those which have escaped antigen targeting. When modified to express matrix metalloprotease 14 (MMP14) enzyme that can digest the ECM, γδ T cells were able to more efficiently migrate in the tumor milieu (112). However, despite being able to kill TNBC cells effectively *in vitro* and showing an improved migration profile, MMP14-engineered γδ T cells could not eliminate TNBC tumors *in vivo* due to down regulation of γδ T cell ligands Fas, MICB and intercellular adhesion molecule 1 (ICAM-1) on breast cancer stem cells. Pre-treatment using zoledronate recovered some cancer stem cell killing by γδ T cells, suggesting that prior activation of γδ T cells may be necessary for TNBC eradication.

#### 3.3.3 Combination therapies

In addition to the aforementioned strategies, supplementing γδ T cell therapy with immune checkpoint inhibitors, such as those targeting PD-1/PD-L1 and CTLA4 pathways (65), or novel cancer stem cell-targeting strategies may further bolster the effectiveness and durability of engineered γδ T anti-tumor responses. Rossi et al. demonstrated that γδ T cells infiltrating follicular lymphoma highly express PD-1 and anti-PD1 blockade consequently increased their cytotoxicity (113).

Another combination treatment that has shown better efficacy is the treatment of locally advanced pancreatic cancer using irreversible electroporation (IRE) with γδ T cells infusion (NCT03180437). The median overall survival of patients treated with IRE alone was 11 months but with γδ T infusions, the overall survival increased to 14.5 months, showing the potential of combination treatment in prolonging patient’s life (53).

Taking advantage of the cross-talk between γδ T cells and other immune cells in the TME, anti-tumor responses in γδ T cell therapies could be further enhanced by boosting the anti-tumor cytotoxicity mediated by other immune cells. For instance, CD137 (4-1BB) co-stimulation with recombinant human CD137L has been shown to increase NKG2D expression on NK cells, which is directly responsible for tumor cell killing (114). An added mechanism of action by these NK cells is the killing of dendritic cells which would otherwise promote inflammation and tumor growth (115).

Other plausible therapies involve targeting the tumor cells within TME, some of which have demonstrated promising preclinical results. These include the use of COX inhibitors which ameliorate the effects of the immunosuppressive TME (116) and celastrol which upregulates death receptor expression on tumor cells (117).

Similarly, patients may also develop resistance with other treatments that could be rescued by co-treatment with γδ T cells. For instance, tyrosine kinase inhibitors (TKIs) have been successful in treating various cancers such as advanced or metastatic renal cell carcinoma, non-small-cell lung cancer and HCC (118–120). However, many patients eventually develop resistance against treatment with TKIs (121). When used alone, γδ T cell therapy also showed some efficacy against these cancers (as summarized in **Table 2**). Therefore, these two complementary therapies potentially add to or synergize with each other in treating cancer patients (48).

## 4 Concluding remarks

γδ T cells are a highly promising immune subset that can be harnessed for “off-the-shelf”, allogeneic immunotherapy (**Figure 1**) and additionally engineered to amplify their anti-tumor efficacy. There exist several hurdles which need to be overcome in order that γδ T cells can be employed as an effective oncotherapy (**Figure 2**). Firstly, the challenge of translating the preclinical finds into clinical trials require extensive knowledge on γδ T cell infiltration and their plasticity within the TME. Learning how to effectively deliver γδ T cells to solid tumor sites by exploiting context-dependent mechanisms which drive γδ T cells to adopt anti- rather than pro-tumor function is absolutely crucial. Secondly, elucidating hitherto unknown ligands that activate and expand specific populations, as defined by TCR usage, of γδ T cells which play important roles in anti-tumor immunity will help to activate γδ T cell in the settings of *in vivo* administration or *ex vivo* expansion. Thirdly, culture conditions and cell source undeniably moulds the γδ T cell final product during the manufacturing process. Identifying the most optimal parameters to adopt in γδ T cell expansion *ex vivo* should be incorporated as part of γδ T cell therapy process development. The relative importance amongst the various γδ T cell subsets and specific depletion of pro-tumorigenic subsets prior infusion should also be considered in the application for γδ T cell therapy. Finally, innovations in modular engineering of γδ T cells and combination strategies will be crucial in improving their *in vivo* anti-tumor cytotoxicity and persistence to prevent tumor relapse whilst minimizing likelihood of detrimental alloreactive responses.

## Author contributions

CW: Conceptualization, Data curation, Investigation, Project administration, Visualization, Writing – original draft, Writing – review & editing. PL: Data curation, Investigation, Visualization, Writing – original draft, Writing – review & editing. AT: Conceptualization, Supervision, Funding acquisition, Writing – review & editing.
