# Supplementary material for: Gamma/delta T cells as cellular vehicles for anti-tumor immunity
Source: Front Immunol. 2024 Jan 11;14:1282758. doi: 10.3389/fimmu.2023.1282758 (PMC10808317; doi:10.3389/fimmu.2023.1282758)
Supplement: Supplementary file 1 [file Table_1.pdf]

Supplemental table. Ongoing and past clinical trials involving direct cellular administration of unmodified and modified γδ T cells, including study outcome (if available).

ClinicalTrials.gov Identifier	Year	Status	Cell type(s) infused	Donor source	Cell source	Modification of cells, if applicable	Trial phase	No. of cells infused per patient	No. of infusions	Condition/disease	Outcome	Reference (if available)	Organiser
	2002	Completed	Enriched in Vγ9Vδ2 T cells (Innacell™; single BrHPP stimulation followed by 2-week expansion in presence of IL-2 in vitro); infused with IL-2	Autologous	PB	nil	1	1 x 10^9, 4 x 10^9 or 8 x 10^9 (no intra-patient dose escalation)	3 (at 3-week interval)	Metastatic RCC	n = 10<br>Efficacy<br>6 SD: 60%<br>4 PD: 40%<br>PFS: 25.7 weeks (5-111 weeks)<br>Safety and toxicity<br>DLT: 1 out of 3 patients treated at 8 x 10^9 cells	(1)	
	2005	Completed	Activated by 2-methyl-3-butenyl-1-pyrophosphate and expansion in the presence of IL-2 until day 14	Autologous	PB	nil	Not applicable	ranged from 5 x 10^6 to 3.6 x 10^9 depending on how much γδ T cells expanded	6-12 for 12 weeks (1 or 2-week interval)	Advanced RCC	n=7<br>Efficacy<br>3 PR: 43%<br>Safety and toxicity<br>No serious adverse events observed.	(2)	
	2006	Completed	Expanded using IL-2 and zoledronate	Autologous	PB	nil	1	1 x 10^7 to 7.2 x 10^9	3 - 12 infusions (2-week interval)	NSCLC	n=10<br>Efficacy<br>3 SD: 30%<br>5 PD: 50%<br>Safety and toxicity<br>No serious adverse events observed.	(3)	

ClinicalTrials.gov Identifier	Year	Status	Cell type(s) infused	Donor source	Cell source	Modification of cells, if applicable	Trial phase	No. of cells infused per patient	No. of infusions	Condition/disease	Outcome	Reference (if available)	Organiser
	2011	Completed	Enriched in Vγ9Vδ2 T cells (zoledronate stimulation followed by 2-week expansion in presence of IL-2 in vitro); infused with zoledronate	Autologous	PB	nil	1	0.5 x 10^7 to 2.8 x 10^9	6-8	Breast cancer, cervical cancer and other solid tumors	n=18<br>Efficacy<br>1 CR: 6%<br>2 PR: 11%<br>3 SD: 17%<br>PR and CR achieved with co-treatment.<br>Safety and toxicity<br>No DLT observed.	(4)	
NCT02418481	2015	Completed	γδ T cells with or without DC-CIK cells	Autologous	PB	nil	1 & 2	Not published	Not published	Breast cancer			Fuda Cancer Hospital, Guangzhou<br>Jinan University Guangzhou
NCT02425735	2015	Completed	Vγ9Vδ2 T cells with or without DC-CIK cells	Autologous	PB	nil	1 & 2	4 × 10^8 cells (1 CCA patient)	1 CCA patient received 8 infusions	Hepatocellular liver cancer (including CCA)	1 case study published (allogeneic).<br>Efficacy<br>Positively regulated peripheral immune functions of the patient, depleted tumor activity, improved quality of life, and prolonged his life span.<br>Safety and toxicity<br>No adverse effects.	(5)	Fuda Cancer Hospital, Guangzhou<br>Jinan University Guangzhou
NCT02425748	2017	Completed	γδ T cells with or without DC-CIK cells	Autologous	PB	nil	1 & 2	Not published	Not published	Non small lung cancer (without EGFR mutation)	No published results.		Fuda Cancer Hospital, Guangzhou<br>Jinan University Guangzhou

ClinicalTrials .gov Identifier	Year	Status	Cell type(s) infused	Donor source	Cell source	Modification of cells, if applicable	Trial phase	No. of cells infused per patient	No. of infusions	Condition/disease	Outcome	Reference (if available)	Organiser
NCT03180437	2017	Completed	Vγ9Vδ2 T cells with or without IRE surgery	Allogeneic	PB	nil	1 & 2	Not published	Single or 6	Locally advanced pancreatic cancer	n=62<br>Efficacy<br>Median OS: 14.5 months compared to 11 months without γδ T infusion<br>Median PFS: 11 months compared to 8.5 months without γδ T infusion<br>Safety and toxicity<br>14 serious adverse events (grade 3 and 4) observed that were likely due to IRE treatment and not γδ T cells	(6)	Fuda Cancer Hospital, Guangzhou<br>Jinan University Guangzhou

ClinicalTrials .gov Identifier	Year	Status	Cell type(s) infused	Donor source	Cell source	Modification of cells, if applicable	Trial phase	No. of cells infused per patient	No. of infusions	Condition/disease	Outcome	Reference (if available)	Organiser
NCT03183206, NCT03183219, NCT03183232	2017	Completed	Vγ9Vδ2 T cells expanded using zoledronate, IL-2, IL-15 and vitamin C for 12-14 days	Autologous	PB	nil	1 & 2	Not published	Not published	Breast cancer, liver cancer & lung cancer, respectively	n=132<br>Efficacy<br>18 patients (13.6%) showed response and prolonged survival<br>Median OS (liver cancer patients): 23.1 months compared to 8.1 months in control group<br>Median OS (lung cancer patients): 19.1 months compared to 9.1 months in control group<br>Safety and toxicity<br>No significant adverse events (immune rejection, GvHD or CRS) observed.	(7)	Fuda Cancer Hospital, Guangzhou<br>Jinan University Guangzhou

ClinicalTrials .gov Identifier	Year	Status	Cell type(s) infused	Donor source	Cell source	Modification of cells, if applicable	Trial phase	No. of cells infused per patient	No. of infusions	Condition/disease	Outcome	Reference (if available)	Organiser
NCT03790072	2018	Completed	Ex vivo expanded Vγ9Vδ2 T cells (OmnImmune®) using zoledronate and IL-2	Allogeneic (matched or haploidentical family donors)	PB	nil	1 & 2	3 cohorts (10^6, 10^7, 10^8 cells/kg)	Single	AML	n=7<br>Efficacy<br>1 CR: 14%<br>1 SD: 14%<br>1 MLFS: 14%<br>Safety and toxicity<br>No DLT and significant adverse effect (GvHD or neurotoxicity) observed. 1 patient suffered possible grade 1 CRS.	(8)	TC BioPharm
NCT04696705	2020	Recruiting	Ex-vivo expanded γδ T cells	Allogeneic (blood-related donor)	PB	nil	Early phase 1	Dose escalation, 1×10^7, 3×10^7, 9×10^7 cells/kg	2 cycles x 2 infusions per cycle (14 day interval)	NHL, PTCL	No published results.		Institute of Hematology & Blood Diseases Hospital<br>Beijing GD Initiative Cell Therapy Technology Co., Ltd.<br>Chinese Academy of Medical Sciences
NCT04702841	2020	Recruiting	CAR γδ T cells	Autologous	PB	CD7 CAR	Early phase 1	0.2-5 × 10^6 cells/kg	Single	R/r $CD7^+$ T cell-derived malignant tumors	No published results.		PersonGen BioTherapeutics (Suzhou) Co., Ltd.<br>Anhui Provincial Hospital

ClinicalTrials .gov Identifier	Year	Status	Cell type(s) infused	Donor source	Cell source	Modification of cells, if applicable	Trial phase	No. of cells infused per patient	No. of infusions	Condition/disease	Outcome	Reference (if available)	Organiser
NCT03533816	2020	Recruiting	Expanded/activated γδ T cell, followed by depletion of ab T-cells (INB-100)	Allogeneic (haploidentical donors)	PB	nil	1	1 x 10^6 cells/kg tested in 3 patients<br>other planned dosed: 3 x 10^6, or 10 x 10^6 cells/kg<br>MTD at expansion phase	Single	AML, CML, ALL, MDS	n=7<br>Efficacy<br>7 CR: 100%<br>PFS: 2.6 - 36 months<br>Safety and toxicity<br>No DLT observed.<br>All patients experienced low grade (1-2) GvHD	https://investors.in8bio.com/news-releases/news-release-details/in8bio-presents-positive-new-inb-100-data-showing-long-term	University of Kansas Medical Center and IN8bio Inc.
NCT04165941	2020	Recruiting	γδ T cells (activated and gene modified) (INB-200)	Autologous	PB	MGMT-gene modified to be drug resistant	1	1 x 10^7	3 cohorts: Single or 3 or 6 infusions	Glioblastoma multiforme	n=8<br>Efficacy<br>Cohort 1 (single dose)<br>PFS: 7.4-11.9 months<br>OS: 9.6-17.7 months<br><br>Cohort 2 (3 doses)<br>PFS: 19.4-23.5 months<br>Safety and toxicity<br>No DLT and serious adverse events (CRS and ICANS) observed. Some grade 1-2 treatment emergent adverse events observed.	(9)<br>https://meetings.asco.org/abstracts-presentations/219950 (presented in Jun 2023)<br>https://investors.in8bio.com/news-releases/news-release-details/in8bio-announces-positive-inb-200-phase-1-data-update	IN8bio Inc.

ClinicalTrials .gov Identifier	Year	Status	Cell type(s) infused	Donor source	Cell source	Modification of cells, if applicable	Trial phase	No. of cells infused per patient	No. of infusions	Condition/disease	Outcome	Reference (if available)	Organiser
NCT04990063	2021	Recruiting	Tumor killer cells: mixed cocultures of NK cells & γδ T cells	Autologous	PB	nil	1	Dose escalation starts at 1×10^8 cells/kg.	After the safety assurance of the initial administration, the next course, up to 8 courses, is resumed and the dose maybe increased subsequently at the discretion of the investigators, or reduced for safety reason.	Advanced NSCLC	No published results.		Tang-Du Hospital Shanghai Biomed-union Biotechnology Co., Ltd.
NCT05015426	2021	Recruiting	γδ T cells (Artificial Antigen Presenting Cell-expanded donor T cells)	Allogeneic	Not stated	nil	1	Dose level -1: 1.0 x 10^6 cells/kg (0.75-1.25 x 10^6 cells/kg) *MTD to be determined. Dose level 1: 5.0 x 10^6 cells/kg (3.75-6.25 x 10^6 cells/kg) Dose level 2: 2.5 x 10^7 cells/kg (1.875-3.125 x 10^7 cells/kg) Dose level 3: 1.0 x 10^8 cells/kg (0.75-1.25 x 10^8 cells/kg) MTD	Single	AML	No published results.		H. Lee Moffitt Cancer Center and Research Institute

ClinicalTrials .gov Identifier	Year	Status	Cell type(s) infused	Donor source	Cell source	Modification of cells, if applicable	Trial phase	No. of cells infused per patient	No. of infusions	Condition/disease	Outcome	Reference (if available)	Organiser
NCT04735471 , NCT04911478	2021	Recruiting	Ex vivo activated and expanded Vδ1 T cells, followed by depletion of ab T cells (ADI-001)	Allogeneic	PB	Anti-CD20 CAR (3H7-CD8 HTM-BBz)	1	Dose escalation:30 x 10^6 cells , 100 x 10^6 cells, 300 x 10^6 cells, 1 x 10^9 cells	Single or multiple infusions	Follicular lymphoma, MCL, MZL, burkitt lymphoma, mediastinal lymphoma, DLBCL, NHL	n=16<br>Efficacy<br>11 CR: 69%<br>1 PR: 6%<br>2 SD: 13%<br>2 PD: 12.5%<br>Safety and toxicity<br>No DLT, GvHD, Grade 3 or higher CRS or ICANS reported.	https://investor.adicetbio.com/news-releases/news-release-details/adicet-bio-reports-positive-data-ongoing-adi-001-phase-1-trial https://www.adicetbio.com/file.cfm/42/docs/adi-001_poster_isct_2022b.pdf	Adicet Bio, Inc
NCT05400603	2022	Recruiting	γδ T cells in combination with dinutuximab, temozolomide, irinotecan and zoledronate (Vδ2 T cells)	Allogeneic	PB	nil	1	Dose escalation: first dose level of 3 x 10^6 cells/kg, second dose level of 3 x 10^6 cells/kg, third dose level of 1 x 10^7 cells/kg, fourth dose level of 3 x 10^7 cells/kg	up to 4 (1-week interval)	R/r neuroblastoma (pediatric)	No published results.		Expression Therapeutics
NCT05653271	2022	Recruiting	Vδ2 T cells (ACE1831) or ACE1831 and obinutuzumab	Allogeneic	PB	anti-CD20 antibody conjugated	1	Not published	Not published	B cell lymphoma, NHL, DLBCL, primary mediastinal large B cell lymphoma, MZL, follicular lymphoma	No published results.		Acepodia Biotech, Inc.
NCT04764513	2021	Recruiting	Ex vivo expanded γδ T cells (expansion from same donors as HSCT)	Allogeneic	PB	nil	1 & 2	Dose escalation, 2×10^6, 1×10^7 and 5×10^7 cells/kg	3 cohorts dose escalation; 5 infusions	Hematological malignancies after allogeneic HSCT: AML, ALL, MDS, lymphoma	No published results.		Chinese PLA General Hospital

ClinicalTrials .gov Identifier	Year	Status	Cell type(s) infused	Donor source	Cell source	Modification of cells, if applicable	Trial phase	No. of cells infused per patient	No. of infusions	Condition/disease	Outcome	Reference (if available)	Organiser
NCT04765462	2021	Recruiting	Ex vivo expanded γδ T cells (expansion from same donors as HSCT)	Allogeneic	Not stated	nil	1 & 2	2x10^6/kg, 1 x10^7/kg to 5x10^7/kg every 2-4 weeks to determine the recommended dose level.	Dosing every 2-4 weeks, number of doses not stated	Malignant solid tumour	No published results.		Chinese PLA General Hospital
NCT05554939	2022	Recruiting	CAR γδ T cells	Allogeneic	PB	anti-CD19 CAR	1 & 2	3 dose groups (2 x 10^6 cells/kg, 6 x 10^6 cells/kg, 1.8 x 10^7 cells/kg)	3	R/r B cell NHL	No published results.		Chinese PLA General Hospital
NCT05886491	2023	Recruiting	Enriched for Vδ1+ γδ T cells (GDX012) after lymphodepleting chemotherapy (fludarabine/cyclophosphamide)	Allogeneic	PB	nil	1 & 2	3 dose groups (weight based dose)	Single infusion, with some patients eligible for second dose	AML	No published results.		Takeda Pharmaceutical Company Limited (acquired GammaDelta Therapeutics Limited)
NCT03849651	2019	Recruiting	TCRαβ-depleted hematopoietic cell transplantation with additional memory cell DLI and selected use of blinatumomab	Allogeneic/haploidentical	PB	nil	2	Not published	Single	ALL, AML, MDS, NK cell Leukemia, Hodgkin lymphoma, NHL, JMML, CML	No published results.		St. Jude Children's Research Hospital
NCT05358808	2022	Recruiting	Vδ2 T cells (TCB-008)	Allogeneic	PB	nil	2	7 x 10^7 or 7 x 10^8 cells	Single	AML	No published results.		TC BioPharm Royal Marsden Hospital London
NCT05686538	2023	Recruiting	Innate donor lymphocyte infusion enriched in NK and γδ T cells	Allogeneic	PB/BM	nil	2 & 3	>10^7 NK cells/kg and >10^6 TCRgd cells/kg	Single	AML, MDS	No published results.		Rigshospitalet
NCT05388305	2022	Recruiting	CAR γδ T cells	Allogeneic	Not stated	anti-CD123 CAR	Not applicable	Not published	Not published	R/r AML	No published results.		Hebei Senlang Biotechnology Inc., Ltd

ClinicalTrials.gov Identifier	Year	Status	Cell type(s) infused	Donor source	Cell source	Modification of cells, if applicable	Trial phase	No. of cells infused per patient	No. of infusions	Condition/disease	Outcome	Reference (if available)	Organiser
NCT05302037	2022	Not yet recruiting	CAR γδ T cells	Allogeneic	PB	NKG2DL-targeting CAR	1	Dose escalation: 1 x 10^7, 1 x 10^8, 3 x 10^8 or 1 x 10^9 per infusion<br>Same Dose: 1 x 10^9	4 (1-week interval)	Advanced solid tumours or haematological malignancies	No published results.		CytoMed Therapeutics Pte Ltd
NCT03939585	2023	Not yet recruiting	NK/γδ T cell-enriched product (donor lymphocytes depleted of TCR-αβ T cells and B cells)	Allogeneic (HLA matched sibling donors or partially matched, related haploidentical donors)	PB	nil	1	Not published	Single	Allogeneic stem cell transplant candidate<br><br>AML, ALL, MDS, MPN, LPD	No published results.		Leland Metheny
NCT04806347	2023	Not yet recruiting	TCRαβ+/CD19+ depleted HSC graft	Allogeneic (closely matched unrelated donors or haploidentical related donors)	PB	nil	1	Not published	Not published	Blood disease	No published results.		University of Wisconsin, Madison
NCT05664243	2023	Not yet recruiting	γδ T cells (DeltEx) (INB-400)	Allogeneic	PB	genetically-modified (drug resistance immunotherapy)	1 & 2	Not published	6 (4-week interval)	Recurrent or newly diagnosed glioblastoma	No published results.		IN8Bio Inc
NCT00562666	2008	Terminated	γδ T cells	Autologous	PB	nil	1	5 x 10^8 or 10 x 10^8	Single (hepatic intra arterial administration)	HCC	No published results.		Rennes University Hospital<br>Innate Pharma
NCT05001451	2021	Terminated (business decision, not related to safety)	Enriched for Vδ1+ γδ T cells (GDX012)	Allogeneic	PB	nil	1	Not published	Single	AML	No published results.		GammaDelta Therapeutics Limited

ClinicalTrials.gov Identifier	Year	Status	Cell type(s) infused	Donor source	Cell source	Modification of cells, if applicable	Trial phase	No. of cells infused per patient	No. of infusions	Condition/disease	Outcome	Reference (if available)	Organiser
NCT05628545	2021	Withdrawn (COVID Pandemic)	γδ T cells (GDKM-100)	Allogeneic	Not stated	nil	1 & 2	3 dose groups (2×10^8 cells/person, 5×10^8 cells/person, 10×10^8 cells/person at 1-3 infusion, 4-6 infusion and 7-9 infusion) or 10 x 10^8 cells/person	up to 9	Advanced HCC	No published results.		Guangdong GD Kongming Biotech LLC
NCT02459067	2015	Terminated	γδ T cells (ImmuniCell®)	Autologous	PB	nil	2	Intra-patient dose escalation to achieve a total dose of 30 x 10^9 γδ T cells.	6 (2-week interval)	Malignant melanoma, NSCLC, RCC	No published results.		TC BioPharm
NCT04700319	2019	Unknown	CAR γδ T cells	Autologous	PB	CD19/CD20 CAR	Early phase 1	0.2-5×10^6 cells/kg	Single	Advanced CD19/CD20^{+} B cell line recurrent or refractory hematological malignancies	No published results.		PersonGen BioTherapeutics (Suzhou) Co., Ltd. Anhui Provincial Hospital
NCT04028440	2019	Unknown	γδ T cells	Autologous	PB	nil	Early phase 1	1~2×10^9 per infusion	2 infusions x 3 (4-week interval)	NHL, r/r B cell NHL, CLL, PTCL	No published results.		Beijing GD Initiative Cell Therapy Technology Co., Ltd.
NCT04518774	2020	Unknown	Ex-vivo expanded γδ T cells	Allogeneic (blood-related donor)	PB	nil	Early phase 1	Dose escalation, 1 x 10^7, 3 x 10^7, 9 x 10^7 cells/kg	3 cycles x 2 infusions per cycle (4-week interval)	HCC	No published results.		Beijing 302 Hospital Chinese Academy of Medical Sciences Beijing GD Initiative Cell Therapy Technology Co., Ltd.
NCT02656147	2017	Unknown	CAR γδ T cells	Allogeneic	Not stated	Anti-CD19-CAR	1	Not published	Not published	Leukemia, lymphoma	No published results.		Beijing Doing Biomedical Co., Ltd.
NCT04008381	2019	Unknown	Ex-vivo expanded γδ T cells	Allogeneic (blood-related donor)	PB	nil	1	Dose escalation, 2 x 10^6, 4 x 10^6, 8 x 10^6 cells/kg	Not published	AML	No published results.		Wuhan Union Hospital, China
NCT04107142	2019	Unknown	CAR γδ T cells	Allogeneic/haploidentical	PB	NKG2DL-targeting CAR	1	3 dose levels: 3 x 10^8, 1 x 10^9, 3 x 10^9	4 (1-week interval)	Colorectal cancer, TNBC, sarcoma, NPC, prostate cancer, gastric cancer	No published results.		CytoMed Therapeutics Pte Ltd

ClinicalTrials .gov Identifier	Year	Status	Cell type(s) infused	Donor source	Cell source	Modification of cells, if applicable	Trial phase	No. of cells infused per patient	No. of infusions	Condition/disease	Outcome	Reference (if available)	Organiser
NCT02585908	2019	Unknown	γδ T cells with or without CIK cells	Autologous	PB	nil	1 & 2	Not published	Not published	Gastric cancer	No published results.		Beijing Doing Biomedical Co., Ltd.
NCT04796441	2020	Unknown	CAR γδ T cells	Allogeneic	PB	anti-CD19 CAR	Not applicable	Not published	Not published	Relapsed AML	No published results.		Hebei Senlang Biotechnology Inc., Ltd
NCT03885076	2018	Unknown	CAR Vd2 T cells	Autologous	PB/BM	anti CD33 CAR	Not applicable (observational study)	Not published	Not published	AML (except M3)	No published results.		Royal Marsden NHS Foundation Trust TC BioPharm

Abbreviations: DC, dendritic cells; CIK, cytokine-induced killer cells; IRE, irreversible electroporation; HSCT, hematopoietic stem cell transplantation; HSC, hematopoietic stem cell; HLA, human leukocyte antigen; PBMC, peripheral blood mononuclear cells; BM, bone marrow; CAR, chimeric antigen receptor; MTD, maximum tolerated dose; HCC, hepatocellular carcinoma; CCA, cholangiocarcinoma; EGFR, epidermal growth factor receptor; NSCLC, non-small cell lung cancer; RCC, renal cell cancer; r/r, relapsed or refractory; NHL, non-Hodgkin lymphoma; PTCL, peripheral T cell lymphoma; AML, acute myeloid leukemia; CML, chronic myeloid leukemia; CLL, chronic lymphocytic leukemia; ALL, acute lymphoblastic leukemia; TNBC, triple-negative breast cancer; MDS, myelodysplastic syndromes; MPN, myeloproliferative neoplasm; LPD, lymphoproliferative disorders; MCL, mantle-cell lymphoma; MZL, marginal zone lymphoma; DLBCL, Diffuse large B cell lymphoma; NPC, nasopharyngeal carcinoma; JMML, Juvenile myelomonocytic leukemia; EGFR, epidermal growth factor receptor; TAC, T cell antigen coupler; CR, complete response; PR, partial response; SD, stable disease; MLFS, morphologic leukemia-free state; PFS, progression-free survival; OS, overall survival; DLT, dose-limiting toxicity; CRS, cytokine release syndrome; GvHD, graft-versus-host disease; ICANS, immune effector cell-associated neurotoxicity syndrome

References

1. Bennouna J, Bompas E, Neidhardt EM, Rolland F, Philip I, Galéa C, et al. Phase-I study of Innacell gammadelta, an autologous cell-therapy product highly enriched in gamma9delta2 T lymphocytes, in combination with IL-2, in patients with metastatic renal cell carcinoma. Cancer Immunol Immunother. 2008 Nov;57(11):1599–609. doi: 10.1007/s00262-008-0491-8

2. Kobayashi H, Tanaka Y, Yagi J, Osaka Y, Nakazawa H, Uchiyama T, et al. Safety profile and anti-tumor effects of adoptive immunotherapy using gamma-delta T cells against advanced renal cell carcinoma: A pilot study. Cancer Immunol Immunother. 2007 Apr;56(4):469–76. doi: 10.1007/s00262-006-0199-6

3. Nakajima J, Murakawa T, Fukami T, Goto S, Kaneko T, Yoshida Y, et al. A phase I study of adoptive immunotherapy for recurrent non-small-cell lung cancer patients with autologous gammadelta T cells. Eur J Cardiothorac Surg. 2010 May 1;37(5):1191–7. doi: 10.1016/j.ejcts.2009.11.051

4. Nicol AJ, Tokuyama H, Mattarollo SR, Hagi T, Suzuki K, Yokokawa K, et al. Clinical evaluation of autologous gamma delta T cell-based immunotherapy for metastatic solid tumours. Br J Cancer. 2011 Sep 6;105(6):778–86. doi: 10.1038/bjc.2011.293

5. Alnaggar M, Xu Y, Li J, He J, Chen J, Li M, et al. Allogenic Vγ9Vδ2 T cell as new potential immunotherapy drug for solid tumor: a case study for cholangiocarcinoma. J Immunother Cancer. 2019 Feb 8;7(1):36. doi: 10.1186/s40425-019-0501-8

6. Lin M, Zhang X, Liang S, Luo H, Alnaggar M, Liu A, et al. Irreversible electroporation plus allogenic Vγ9Vδ2 T cells enhances antitumor effect for locally advanced pancreatic cancer patients. Signal Transduct Target Ther. 2020 Oct 23;5(1):1–9. doi: 10.1038/s41392-020-00260-1

7. Xu Y, Xiang Z, Alnaggar M, Kouakanou L, Li J, He J, et al. Allogeneic Vγ9Vδ2 T-cell immunotherapy exhibits promising clinical safety and prolongs the survival of patients with late-stage lung or liver cancer. Cell Mol Immunol. 2020 Sep 16;18(2):427–39. doi: 10.1038/s41423-020-0515-7

8. Vydra J, Cosimo E, Lesný P, Wanless RS, Anderson J, Clark AG, et al. A Phase I Trial of Allogeneic γδ T Lymphocytes From Haploidentical Donors in Patients With Refractory or Relapsed Acute Myeloid Leukemia. Clin Lymphoma Myeloma Leuk. 2023 May 1;23(5):e232–9. doi: 10.1016/j.clml.2023.02.003

9. Nabors LB, Lamb LS, Beelen MJ, Pillay T, Haak M ter, Youngblood S, et al. Phase 1 trial of drug resistant immunotherapy: A first-in-class combination of MGMT-modified γδ t cells and temozolomide chemotherapy in newly diagnosed glioblastoma. Journal of Clinical Oncology. 2021 May 28;39(15_suppl):2057–2057. doi: 10.1200/jco.2021.39.15_suppl.2057
